# The relationship between mean platelet volume and diabetic retinopathy: a systematic review and meta-analysis

**DOI:** 10.1186/s13098-019-0420-3

**Published:** 2019-03-12

**Authors:** ShuaiFei Ji, Jie Zhang, XiuDe Fan, XiQiang Wang, XiaoNa Ning, BaBo Zhang, Heng Shi, Hong Yan

**Affiliations:** 1 Department of Ophthalmology, Tangdu Hospital, the Fourth Military Medical University, Xian, 710038 Shaanxi People’s Republic of China; 2 Department of Infectious Diseases, First Affiliated Hospital of Xian Jiaotong University, Xian, 710061 Shaanxi People’s Republic of China; 3 Department of Cardiovascular Medicine, First Affiliated Hospital of Xian Jiaotong University, Xian, 710061 Shaanxi People’s Republic of China; 4 Department of Ophthalmology, Xi’an No. 4 Hospital, Shaanxi Eye Hospital, Affiliated Guangren Hospital School of Medicine, Xi’an Jiaotong University, Xian, 710004 Shaanxi China

**Keywords:** Mean platelet volume, Diabetic retinopathy, Meta-analysis

## Abstract

**Background:**

Diabetic retinopathy (DR) is one of the most common diseases causing blindness in the world, and most patients are already in advanced stage. Recent years, many studies reported mean platelet volume (MPV) may be associated with development of DR, but there was no consistent conclusion reached.

**Methods:**

Literature was retrieved by formally searching PubMed, Embase, Cochrane library and Scopus and by hand searching of reference lists of related articles. Finally, a total of 14 literatures included, and Review manager 5.3 and STATA 14.0 statistical software were utilized for processing.

**Results:**

Meta-analysis showed that MPV values in DR were significantly higher than health controls [SMD (95% CI) = 0.92 (0.60–1.24)] and type 2 diabetes mellitus without diabetic retinopathy (T2DM without DR) [SMD (95% CI) = 0.36 (0.19–0.53)]. Subgroup analysis indicated that MPV level in proliferative diabetic retinopathy (PDR) patients was higher than T2DM without DR patients [SMD (95% CI) = 0.48 (0.28, 0.68)], but this difference didn’t appear in non-proliferative diabetic retinopathy (NPDR).

**Conclusions:**

The study demonstrated that increased MPV level was significant associated with the development of DR, and it might reflect the severity of DR, which could be provided to monitor development and progression of DR clinically.

## Background

Type 2 diabetes mellitus is a common metabolic disease with all kinds of microvascular diseases occurring. Diabetic retinopathy (DR), a kind of microvascular lesions occurring in fundus, accounts for 40 percent of diabetics over 40 years of age [1], and is the main cause of impaired vision and even blindness in diabetics [2]. The pathogenesis of diabetic retinopathy is not clear, relevant studies have shown that it may be associated with local microvascular injury and microcirculation disorders, and improving blood circulation of the retina effectively before or early in the emergence of DR may prevent it from developing [3, 4]. The diagnosis of diabetic retinopathy depends on fundus examination, but it’s not good for routine screening, and the patient’s compliance is poor. Therefore, it is especially important to find simple detection methods. Microthrombus formation caused by microcirculation changes is a pathogenic factor, in which platelet plays an important role [5, 6]. Mean platelet volume (MPV) reflects the average size and function of platelet in a person’s blood sample, of which the relationship with acute myocardial infarction and coronary artery has been shown [7, 8]. As parameters of platelet, high level MPV might be associated with increased thrombotic potential [9], which might participate in development of DR. However, conflicting data are available on MPV in DR, and there hasn’t been a systematic review to assess the relationship.

This study aims to assess and quantify differences in MPV comparing subjects with DR, type 2 diabetes mellitus without diabetic retinopathy (T2DM without DR) and control group, for exploring the relationship between MPV and DR.

## Methods

### Literature search

Literature was retrieved by formal search of electronic databases (PubMed, Embase, Cochrane library and Scupos) and by hand searching of reference lists of related articles. These computer searches were limited to English language articles from the beginning of building database to December 2017, and Chinese language articles must be published on medline. The following keywords were used for searching: ‘‘diabetic retinopathy’’ AND ‘‘mean platelet volume’’. The retrieval strategy of Pubmed as follow: (((((Diabetic Retinopathies [Title/Abstract] OR Retinopathies, Diabetic [Title/Abstract] OR Retinopathy, Diabetic[Title/Abstract])) OR diabetic retinopathy[Title/Abstract]) OR “Diabetic Retinopathy”[Mesh])) AND ((((Mean Platelet Volumes[Title/Abstract] OR Platelet Volume, Mean[Title/Abstract] OR Platelet Volumes, Mean[Title/Abstract] OR Volume, Mean Platelet[Title/Abstract] OR Volumes, Mean Platelet[Title/Abstract])) OR mean platelet volume[Title/Abstract]) OR “Mean Platelet Volume”[Mesh]).

### Selection criteria

The inclusion criteria were as follows: (1) published literature related to the association of MPV level with DR; (2) independent case–control studies or cross-section studies using either a hospital-based or a population-based design; (3) the original studies must provide the number of each group and the mean and standard of MPV. Excluded criteria: (1) duplicated data; (2) the original data could not be extracted.

### Data extraction and quality assessment

Two authors (SF Ji and XD Fan) independently extracted the original data. Disagreement was resolved by discussion. If the two authors could not reach a consensus, the result was reviewed by a third author (XN Ning). The extracted data were consisted of the follow items: the first author’s name, publication year, population (Ethnicity), methods, study design, matching criteria, sex, total number of cases and controls, and age (years). Study quality was assessed by the Newcastle–Ottawa scale (NOS), which uses a ‘‘star’’ rating system to judge the quality of all observational studies. The NOS ranges between zero (worst) up to nine stars (best) and studies with a score equal to or higher than seven were considered to be of high quality. Two investigators (SF Ji and XD Fan) independently assessed the quality of the included studies, and the results were reviewed by a third investigator (J Zhang). Disagreement was resolved by discussion.

### Statistical analysis

We utilized Review manager 5.3 and Stata 14.0 software to perform the meta-analysis in the present study. Heterogeneity among studies was assessed by I^2^ statistic, P < 0.10 and I^2^ > 50% indicated evidence of heterogeneity. If heterogeneity existed among the studies, the random effects model was used to estimate the pooled standard mean difference (SMD). Otherwise, the fixed effects model was adopted. The standard mean difference (SMD) and corresponding 95% confidence interval (CI) were utilized to assess the associations. The potential publication bias was investigated using Egger’s test and Funnel plot. Egger’s test (P < 0.05) was also considered to be representative of statistically significant publication bias, which was conducted with the Stata14.0 software. Subgroup analysis about study design, location, quality and DR sub-type were performed to further explore the heterogeneity and clinical significance.

## Results

### Study characteristics

**Fig. 1:**
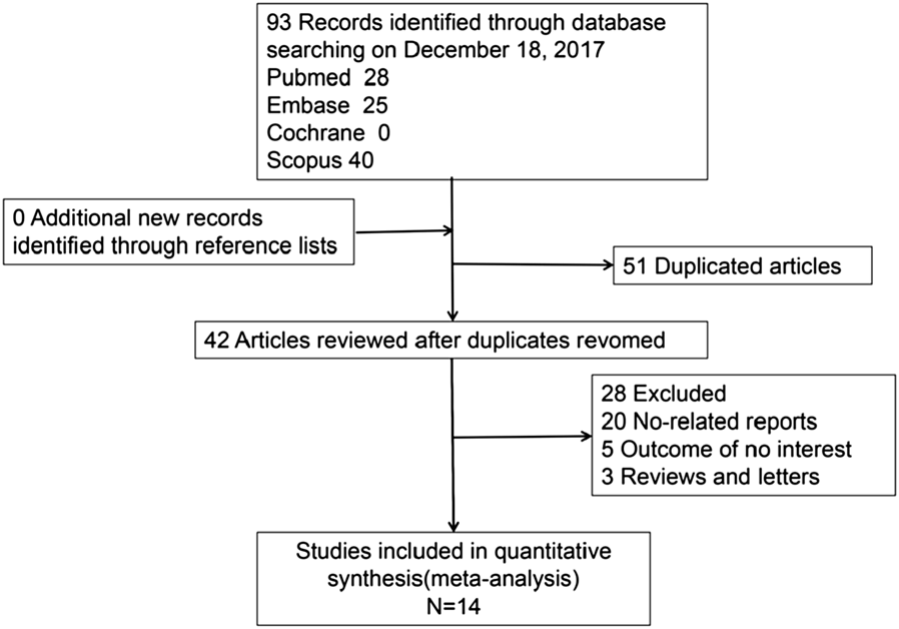
Flow diagram for literature selection

We retrieved a total of 98 studies. After duplicates were removed, only 42 full-text studies were evaluated. After exclusion of review and no-related articles, a total of 14 studies [10–23] were included in the final meta-analysis according to the inclusion criteria, including 2 cross-section studies [10, 14] and 11 case control studies [11–13, 15–23]. There are 1252 cases in the DR group, 1359 cases in T2DM without DR group and 1133 cases in control group. Table 1 shows the characteristics of included studies. Figure 1 shows the process of literature selection. As for the application of anticoagulation methods, ethylenediaminetetraacetic acid (ETDA) was used in 9 literature [11, 13, 15, 16, 18–22], and 5 literature reported the collection and measurement time [18–22]. One literature using citrate [23] and 4 literature have not specifically reported [10, 12, 14, 17].

**Table 1:** Characteristics of included studies

Authors	Location, year	DR		T2DM without DR		Control		-Tubes	NOS
		N	MPV	N	MPV	N	MPV		
Yilmaz et al.	Turkey, 2016	174	8.1 ± 0.83	88	7.81 ± 0.76	85	7.42 ± 0.68	EDTA	9
Ateş et al.	Turkey, 2009	90	7.96 ± 0.76			30	7.52 ± 1.01	EDTA	9
Dindar et al.	Turkey, 2013	24	11.26 ± 1.08	47	10.68 ± 1.68	50	10.23 ± 1.01	EDTA	9
Citirik et al.	Turkey, 2015	97	8.08 ± 0.71	43	7.94 ± 0.63	40	7.74 ± 0.78	EDTA	8
Demirtas et al.	Turkey, 2015	67	9.54 ± 0.88	240	9.2 ± 0.92			NR	8
Tetikoglu et al.	Turkey, 2016	136	8.71 ± 0.82	63	8.51 ± 1	76	8.32 ± 0.9	NR	7
Müberra et al.	Turkey, 2016	120	9.6 ± 1	158	9.7 ± 1.2	107	9.3 ± 1	EDTA	8
Gungor et al.	Turkey, 2016	52	9.3 ± 1	50	8.8 ± 1.1	50	8.3 ± 0.6	EDTA	9
Zhong et al.	China, 2011	200	10.09 ± 0.92			100	9.46 ± 0.93	NR	7
Li et al.	China, 2016	47	10.72 ± 1.57	52	10.39 ± 0.9	48	9.75 ± 0.89	EDTA	8
Zhou et al.	China, 2016	51	10.4 ± 1.1	328	10 ± 1.1	96	9.1 ± 0.8	NR	8
Radha et al.	India, 2016	14	9.2 ± 0.61	30	8.39 ± 0.68	100	8.02 ± 0.86	EDTA	8
Buch et al.	India, 2017	80	11.4 ± 1.96	162	9.91 ± 1.97	200	8.48 ± 1.01	EDTA	6
Papanas et al.	Greece, 2004	167	15.8 ± 1.3	98	10.9 ± 1.1	151	7.1 ± 1.2	Citrate	6

*DR* diabetic retinopathy, *T2DM without DR* type 2 diabetic mellitus without DR, *N* number of subjects, *MPV* mean platelet volume, *NOS* Newcastle–Ottawa scale, *EDTA* ethylenediaminetetraacetic acid, *NR* no report

### Meta-analysis

**Fig. 2:**
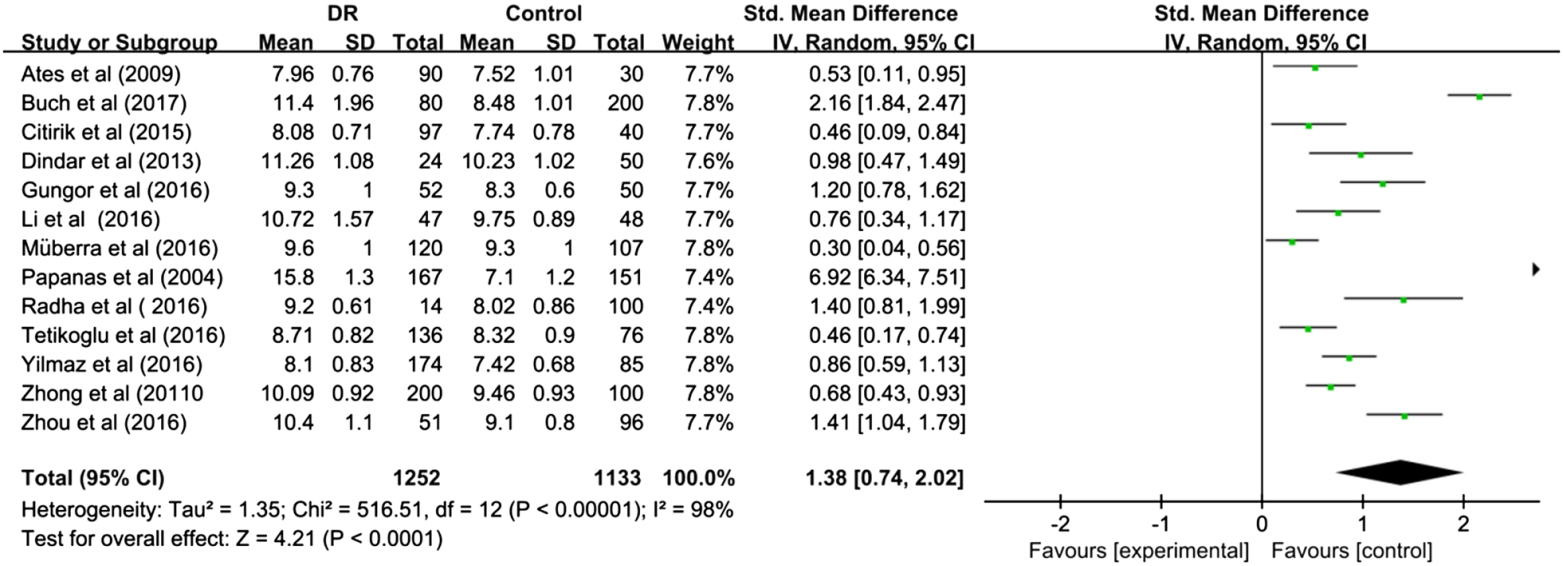
Meta-analysis for mean platelet volume in DR and control

**Fig. 3:**
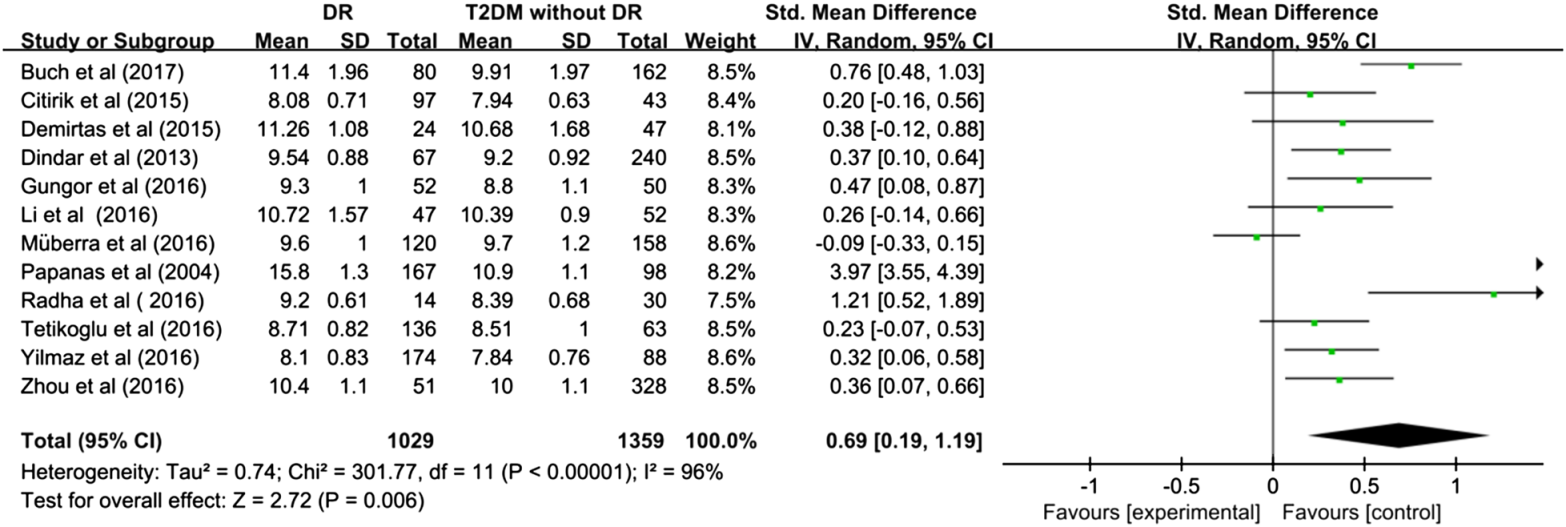
Meta-analysis for mean platelet volume in DR and T2DM without DR

The pooled SMD estimate showed that significant higher value of MPV in DR compared to control group [SMD (95% CI) = 1.38 (0.74, 2.02)] (Fig. 2) and T2DM without DR [SMD (95% CI) = 0.69 (0.19, 1.19)] (Fig. 3). I^2^ test indicated that the heterogeneity was that I^2^ = 98% (P < 0.00001) and I^2^ = 96% (P < 0.00001) respectively, therefore, given the significance, the random-effects model was applied to perform meta-analysis.

### Subgroup analysis

To further explore the origin of heterogeneity and the clinical significance of MPV in assessing severity of DR, subgroups analysis about study design, quality, location and DR sub-type were performed. Unfortunately, we discovered heterogeneity of subgroup was generally high in the comparison to DR and control, low-quality studies in particular, which mean that the origin of heterogeneity was unclear. In the comparison to DR and T2DM without DR, we concluded that study design and low-quality studies generated heterogeneity obviously. Finally, we further explored the association between MPV level and DR type. We found, in terms of MPV level, both NPDR and PDR were significantly higher than control group [SMD (95% CI) = 0.41 (0.16, 0.65) P = 0.81 (0.48, 1.14)], PDR was higher than T2DM without DR [SMD (95% CI) = 0.48 (0.28, 0.68), P = 0.349] and NPDR [SMD (95% CI) = 0.41 (0.17, 0.64)], while NPDR was no difference with T2DM without DR [SMD (95% CI) = 0.04 (− 0.16, 0.24)]. We take gap in time between collection and measuring MPV-60 min to divide into subgroups, and the results showed that (Table 2), when ≤ 60 min, DR vs control, SMD = [0.39 (0.20–0.58), I^2^ = 0%], DR vs T2DM without DR, SMD = [0.02 (− 0.25 to 0.30), I^2^ = 43%], obviously the latter results no statistical significance. Then, when > 60 min, DR vs control, SMD = [0.99 (0.67–1.31), I^2^ = 41%], DR vs T2DM without DR, SMD = [0.37 (0.15–0.58), I^2^ = 0%], no significant difference appeared. Therefore, study design, low-quality articles, DR type and gap in time between collection and measuring MPV were the sources of heterogeneity, and high MPV level might reflect the severity of DR.

**Table 2:** Subgroup analysis of the relation between MPV and DR patients

Subgroup	Study	No. of studies	SMD	95% CI	Heterogeneity	
					P value	I^2^ (%)
DR vs control						
Location	Turkey	7	0.66	0.42–0.90	= 0.003	70
	China	3	0.94	0.49–1.40	= 0.005	81
	India	2	1.82	1.09–2.56	= 0.003	70
Study quality	High	11	0.79	0.57–1.01	< 0.0001	76
	Low	2	4.53	− 0.14 to 9.21	< 0.00001	99
DR vs T2DM without DR						
Location	Turkey	7	0.24	0.09–0.40	= 0.11	42
	China	2	0.33	0.09–0.56	= 0.68	0
	India	2	0.87	0.49–1.25	= 0.23	30
Study design	Case–control	10	0.76	0.17–1.35	< 0.00001	97
	Cross-sectional	2	0.37	0.11–0.62	= 0.95	0
Study quality	High	10	0.30	0.15–0.45	= 0.03	51
	Low	2	2.36	− 0.79 to 5.51	< 0.00001	99
DR sub-type	NPDR vs control	4	0.41	0.16–0.65	= 0.182	38.3
	PDR vs control	4	0.81	0.48–1.14	= 0.031	66.1
	NPDR vs T2DM without DR	3	0.04	− 0.16 to 0.24	= 0.642	0
	PDR vs T2DM without DR	3	0.48	0.28–0.68	= 0.349	5.1
	PDR vs NPDR	4	0.41	0.17–0.64	= 0.193	36.6
Intervals^a^						
≤ 60 min	DR vs control	3	0.39	0.20–0.58	= 0.60	0
	DR vs T2DM without DR	2	0.02	− 0.25 to 0.30	= 0.18	43
> 60 min	DR vs control	2	0.99	0.67–1.31	= 0.19	41
	DR vs T2DM without DR	2	0.37	0.15–0.58	= 0.53	0

*MPV* mean platelet volume, *DR* diabetic retinopathy, *T2DM without DR* type 2 diabetic mellitus without diabetic retinopathy, *NPDR* non-proliferative diabetic retinopathy, *PDR* proliferative diabetic retinopathy, *SMD* standard mean difference, *CI* confidence interval

^a^Intervals of MPV collection and measurement

### Sensitive analysis

The contribution of each study to the pooled estimate was performed in order to assess the sensitivity analysis (Table 3). It was noteworthy that Papanas et al. [23] might cause heterogeneity, which was excluded at a time and recalculated the pooled result. After that, the heterogeneity in DR compared to T2DM without DR was decreased significantly to 66%. Meanwhile, the result was also decreased [SMD (95% CI) = 0.36 (0.19, 0.53)]. Further analysis revealed that the biggest difference between the study on Papanas et al. [23]. and other included literatures lies in the different anticoagulants used in the collection of platelet sample tubes. Only Papanas et al. [23] used citrate and the results didn’t change significantly after excluding Papanas et al. [DR vs control, SMD = 0.92 (0.60–1.24), I^2^ = 90%, DR vs T2DM without DR, SMD = 0.36 (0.19–0.53), I^2^ = 66%], which were considered as final results. Finally, the results of two comparisons exhibited that our meta-analysis was reliable, without inverse changes appearing.

**Table 3:** Sensitivity analysis (leave-one-out approach to) for MPV in DR

Study	SMD	95% CI	P value	I^2^ (%)
DR vs control				
Ates et al.	1.45	0.76–2.13	< 0.00001	98
Buch et al.	1.31	0.65–1.98	< 0.00001	98
Citirik et al.	1.46	0.77–2.14	< 0.00001	98
Dindar et al.	1.41	0.73–2.09	< 0.00001	98
Gungor et al.	1.39	0.71–2.08	< 0.00001	98
Li et al.	1.43	0.74–2.12	< 0.00001	98
Müberra et al.	1.47	0.77–2.17	< 0.00001	98
Papanas et al.	0.92	0.60–1.24	< 0.00001	90
Radha et al.	1.38	0.70–2.05	< 0.00001	98
Tetikoglu et al.	1.46	0.76–2.16	< 0.00001	98
Yilmaz et al.	1.42	0.71–2.14	< 0.00001	98
Zhong et al.	1.44	0.72–2.16	< 0.00001	98
Zhou et al.	1.38	0.68–2.07	< 0.00001	98
DR vs T2DM without DR				
Buch et al.	0.69	0.13–1.24	< 0.00001	97
Citirik et al.	0.74	0.20–1.28	< 0.00001	97
Demirtas et al.	0.72	0.19–1.25	< 0.00001	97
Dindar et al.	0.72	0.17–1.28	< 0.00001	97
Gungor et al.	0.71	0.17–1.25	< 0.00001	97
Li et al.	0.73	0.20–1.27	< 0.00001	97
Müberra et al.	0.77	0.23–1.31	< 0.00001	96
Papanas et al.	0.36	0.19–0.53	= 0.001	66
Radha et al.	0.65	0.13–1.17	< 0.00001	97
Tetikoglu et al.	0.74	0.19–1.29	< 0.00001	97
Yilmaz et al.	0.73	0.17–1.29	< 0.00001	97
Zhou et al.	0.72	0.17–1.28	< 0.00001	97

### Publication bias

**Fig. 4:**
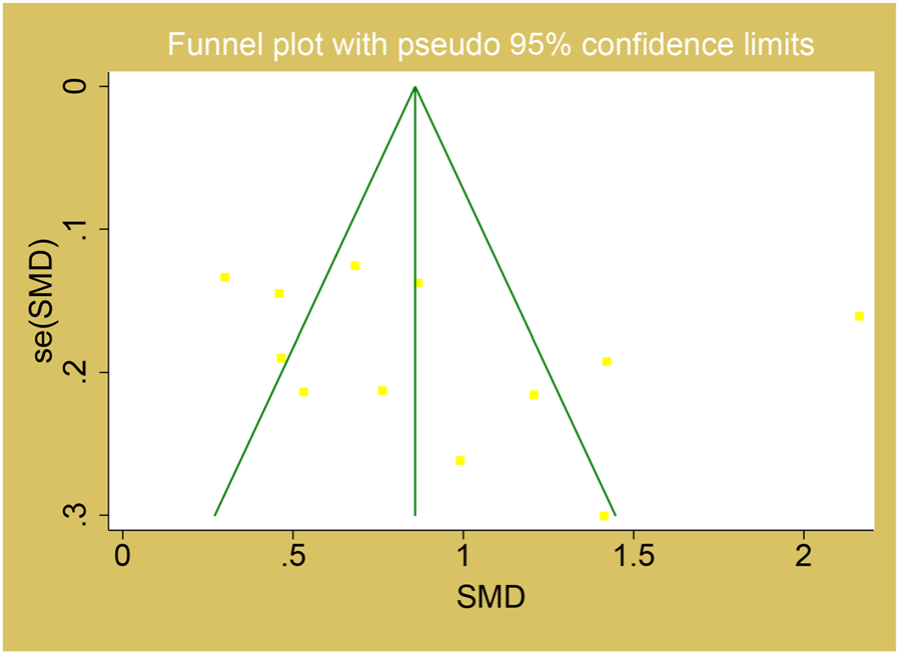
Funnel plot for MPV in the comparison of DR and control

**Fig. 5:**
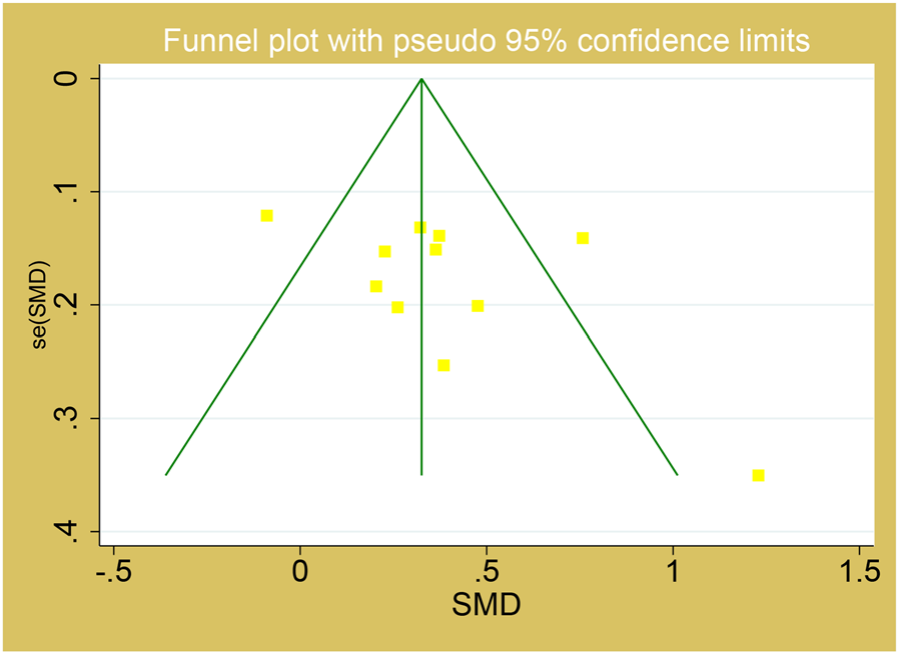
Funnel plot for MPV in the comparison of DR and T2DM without DR

The publication bias was evaluated using funnel plot and egger test. There was no publication bias existing in MPV level with DR compared to control group (Egger’s P = 0.36) and T2DM without DR group (Egger’s P = 0.15). The funnel plots were shown in Figs. 4 and 5 respectively.

## Discussion

To our knowledge, this is the first study that systematically reviews and summarizes through a meta-analysis to explore the relationship between platelet parameters and DR. Our results evidenced statistically significantly higher values of MPV in DR compared with T2DM without DR and health.

Platelets were one of the causes of capillary nonperfusion in diabetes. Qualitative abnormalities and activation of platelet in DM have been reported [24], which has a close relationship with insulin resistance, hyperglycemia, and dyslipidemia [25, 26]. Larger platelets are more active because of elevated prothrombic contents, such as thromboxane A2, thromboxane B2, platelet factor 4, serotonin, and platelet-derived growth factor (PDGF) [27]. Some studies revealed that platelet participated in development of DR by thrombogenesis with microvascular lesions [12], and so far, the specific mechanisms of platelets in DR focuses on platelet-derived growth factor (PDGF), which is released from platelets. Eng et al. [28] reached a conclusion that pericyte loss caused by PDGF-B may also be a causal pathogenic event in human DR. Yokota et al. [29] found that hyperglycemia can increase PDGF-B levels in the retina, which mediated via PDGF-β receptors in part by protein kinase C (PKC) activation to upregulate expression of an essential factor endothelin-1 (ET-1) participated in pathophysiology of DR. Geraldes et al. [30] further found hyperglycemia persistently activated PKCδ and p38α MAPK to increase the expression of a novel target, Scr homology-2 domain containing phosphatase-1 (SHP-1), leading to PDGF receptor-β dephosphorylation and actions, and increased pericyte apoptosis, independent of NF-κB, and Chen et al. [31] reached the similar conclusion. Praidou et al. [32] discovered the correlation between PDGF and NPDR, and topical ketorolac tromethamine to treat PDR caused PDGF levels to decrease [33]. Therefore, platelets play an important role in formation of DR. However, clopidogrel (the selective antiplatelet drug), did not prevent neuronal apoptosis, glial reactivity, capillary cell apoptosis, or acellular capillaries in retinas of diabetic rats [34], suggesting that platelet do not initiate the pathology of early diabetic retinopathy.

MPV is positively correlated with platelet adhesion and aggregation, the higher level MPV, the higher rate and stronger function of platelets. Recent research found that MPV was strongly and independently associated with the presence and severity of diabetes [35], and there were great significance of cardio-vascular complications in diabetes mellitus [36], which may be associated with osmotic change [37]. Taurine is a key compound in osmoregulation, which plays an important role in maintaining cell volume [38, 39]. Taurine is found in high concentration in platelets [40], the level of which in platelets decreases during diabetes, and a clinical study involving oral administration of taurine to diabetes patients showed that the platelet hyperaggregation could be normalised [41, 42]. Therefore, increased MPV in patients with diabetic retinopathy may be associated with decreased taurine levels. Furthermore, a study in diabetic rats with an aldose reductase inhibitor showed that polyol pathway activity is involved in the hyperaggregability of platelets [43]. High plasma glucose could increase the intracellular glucose level, which leads to abnormal activation of aldose reductase, a key enzyme in the polyol pathway, reducing glucose to sorbitol [44, 45]. Sorbitol is a polyhydroxy alcohol, hydrophilic, not easy to penetrate the cell membrane, accumulating intracellularly with possible osmotic consequences [44]. The accumulation of sorbitol causes depletion of other osmolytes, such as taurine, causing dysfunction of cell volume regulation [46]. Retinal microvascular lesion of DR is characterized by thickening and microthrombosis of capillary base, and platelet dysfunction has an important influence on development of microvascular complications. The larger MPV, the more likely formation of thrombosis, and in other hand, vascular endothelial injury triggers platelet adhesion and aggregation to accelerate thrombosis. Subgroup analysis exhibited MPV level in NPDR was no difference with T2DM without DR, but in PDR was higher than both of them, which was consistent with theory that platelets do not initiate the pathology of early DR. We reached conclusions that DR grade resulted in the heterogeneity and MPV level was also upregulated in higher DR severity. In addition, in the pooled analysis of MPV, we discovered the heterogeneity was decreased significantly after excluding Papanas et al. [23]. What is special about this article is that only citrate is used in it, while other included articles using EDTA. Citrate is mainly used for hemostasis test and blood sedimentation test. Because its toxicity is small, also used in blood transfusion maintenance fluid. The anticoagulant mechanism is that citrate forms a soluble chelate with calcium ions in blood to prevent blood coagulation. However, the coagulation time of plasma from different sources of thrombin reagents can vary greatly for the same normal person or patient. The results did not change significantly after excluding it, so we took the results of exclusion. We hold the view that the reason why T2DM without DR patients of it might arise from other potential complications affecting real result of MPV, such as nephropathy [15]. However, the final result of MPV didn’t change, which suggested the reliability of our results. There were some articles reporting the relationship between other hematological indicators and diabetic retinopathy, such as NLR [47, 48]. A latest systematic review reported, similar to MPV, higher level NLR appears in DR compared to control and T2DM without DR [49], which may be useful for monitoring DR when combined with MPV.

Of course, there were some limits in our article. First of all, the definition and diagnosis of T2DM without DR and DR were not consistent completely. All of our included studies were case–control or cross-sectional studies, so we couldn’t suppress interference of other non-matched factors. Only English language was included in this meta-analysis, so some eligible studies, which were unpublished or reported in other languages, were likely missed. The intervals of MPV collection and measurement are not completely consistent. Some diabetes-related factors, such as glycaemic control, duration of diabetes and kidney complications, were difficult to be corrected. In addition, many studies have reported that drugs, including statins and metformin, also could affect platelets [50–54], but none of the included articles mentioned patients’ medication status. All the factors mentioned above may be sources of heterogeneity, which should be paid attention to in future research design.

## Conclusions

Fortunately, higher values of MPV in DR vs T2DM without DR were exhibited, hence, we concluded that platelets have a closed relationship with DR. MPV is easily accessible platelet volume parameters and reflect function of platelet, so it will be of great significance if we can monitor the development and progression of DR with it. Given the significance of MPV in DR grade, we need to attach importance to MPV in the development of DR. Taking account of the limits in this study, more rigorous and high-quality researches need to be implemented to further confirm our conclusions.

## Abbreviations

MPV: mean platelet volume
DR: diabetic retinopathy
T2DM without DR: type 2 diabetes mellitus without diabetic retinopathy
PDR: proliferative diabetic retinopathy
NPDR: non-proliferative diabetic retinopathy
NOS: Newcastle–Ottawa scale
SMD: standard mean difference
CI: confidence interval
